# Acromioclavicular joint reconstruction with coracoacromial ligament transfer using the docking technique

**DOI:** 10.1186/1471-2474-10-6

**Published:** 2009-01-14

**Authors:** Peter J Millett, Sepp Braun, Reuben Gobezie, Iván H Pacheco

**Affiliations:** 1 Steadman Hawkins Research Foundation, 181 West Meadow Drive, Suite 1000, Vail, CO, USA; 2 The Case Shoulder & Elbow Service, Case Western Reserve University School of Medicine, 10900 Euclid Ave, Cleveland, OH, USA; 3 Harvard Shoulder Service, Harvard Medical School, Brigham and Women's Hospital, 75 Francis St, Massachusetts General Hospital, Boston, MA, USA

## Abstract

**Background:**

Symptomatic Acromioclavicular (AC) dislocations have historically been surgically treated with Coracoclavicular (CC) ligament reconstruction with transfer of the Coracoacromial (CA) ligament. Tensioning the CA ligament is the key to success.

**Methods:**

Seventeen patients with chronic, symptomatic Type III AC joint or acute Type IV and V injuries were treated surgically. The distal clavicle was resected and stabilized with CC ligament reconstruction using the CA ligament. The CA ligament was passed into the medullary canal and tensioned, using a modified 'docking' technique. Average follow-up was 29 months (range 12–57).

**Results:**

Postoperative ASES and pain significantly improved in all patients (p = 0.001). Radiographically, 16 (94%) maintained reduction, and only 1 (6%) had a recurrent dislocation when he returned to karate 3 months postoperatively. His ultimate clinical outcome was excellent.

**Conclusion:**

The docking procedure allows for tensioning of the transferred CA ligament and healing of the ligament in an intramedullary bone tunnel. Excellent clinical results were achieved, decreasing the risk of recurrent distal clavicle instability.

## Background

**Figure 1:**
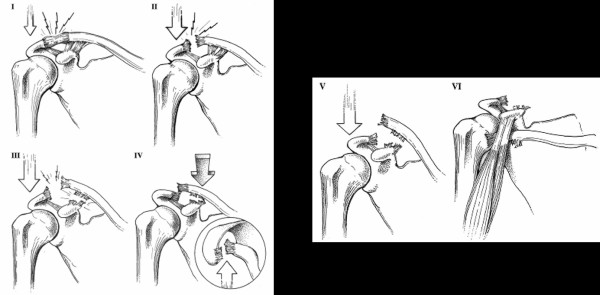
**Classification of acromioclavicular joint injuries type I-VI and type V–VI**. Reprinted with permission: *OPERATIVE TECHNIQUES IN SPORTS MEDICINE, V12(1): 35–42, Millett PJ et al: "Acromioclavicular Joint Instability...". ^© ^2004 Elsevier Inc*.

Injuries from sporting activities account for 25–50% of all acromioclavicular (AC) separations. [1-5] These injuries are the second most common type of dislocation to occur around the shoulder girdle at an overall incidence of almost 4 per 100,000 in the general population. [1-7] The vast majority of AC separations (grades I, II, III) do very well with conservative treatment (Figure 1).[6,7] Grades IV-VI are uncommon and are usually the result of a very high-energy injury which may need surgical repair (Figure 1).[8] Yet, standard method for treating this common injury is still lacking, with more than sixty different surgical reconstruction techniques described.[7]

Most techniques for AC joint reconstruction involve reconstructing the coracoclavicular (CC) ligament with some modification of the Weaver-Dunn technique, which transfers the CA ligament to the distally resected end of the clavicle.[9,10] By this means the clavicle is reduced and the joint is functionally reconstructed. Biomechanical studies show that these reconstructions have structural properties markedly different from those of the intact CC ligaments. [11-13] Additionally, other studies show that the current surgical reconstructions either fail to restore stability or over reduce the AC joint.[14,15] Of late there has also been interest in anatomic CC ligament reconstructions with biomechanical data that supports these techniques.[1] The docking technique was originally described by Rohrbough and Altchek [16,17] for elbow MCL reconstruction allowing optimal tensioning of the reconstructed ligament into a bone tunnel. In most series the method of tensioning the transferred CA ligament is not well defined. In our series we use a similar "docking" of the transferred CA ligament into the clavicle in a systematic way that achieves optimal tension reproducibly. The presented docking technique is a non-anatomic reconstruction of the AC joint. The purpose of this study was to describe the details of our surgical technique and to present the obtained clinical results. We believe this study will serve as a comparison group for other upcoming techniques (e.g. anatomical or arthroscopically-assisted).

## Methods

From 2001 to 2004, 17 consecutive patients with chronic, symptomatic AC joint type III or acute types IV and V injuries were treated by the senior author (PJM) with surgical reconstruction of the CC ligaments. At the time of the IRB approved study, these were considered the standard indications for operative treatment of an AC injury. Patients were identified retrospectively for this study based on the procedure performed. IRB approval was given by the IRB of the Brigham and Women's hospital, Boston, MA. The surgical technique consisted of a modification of the Weaver-Dunn technique with resection and reduction of the distal clavicle, transfer of the CA ligament, and augmentation of the fixation with PDS sutures.[18] In our modified AC joint reconstruction, we augment the transferred CA ligament with a 9-strand PDS braid.[18,19] Early in the series, three patients had additional augmentation with a palmaris longus autograft. The mean follow-up was 29 months (range, 12–57 months). There were 12 men and five women, with a mean age of 44 (range, 22–70).

**Figure 2:**
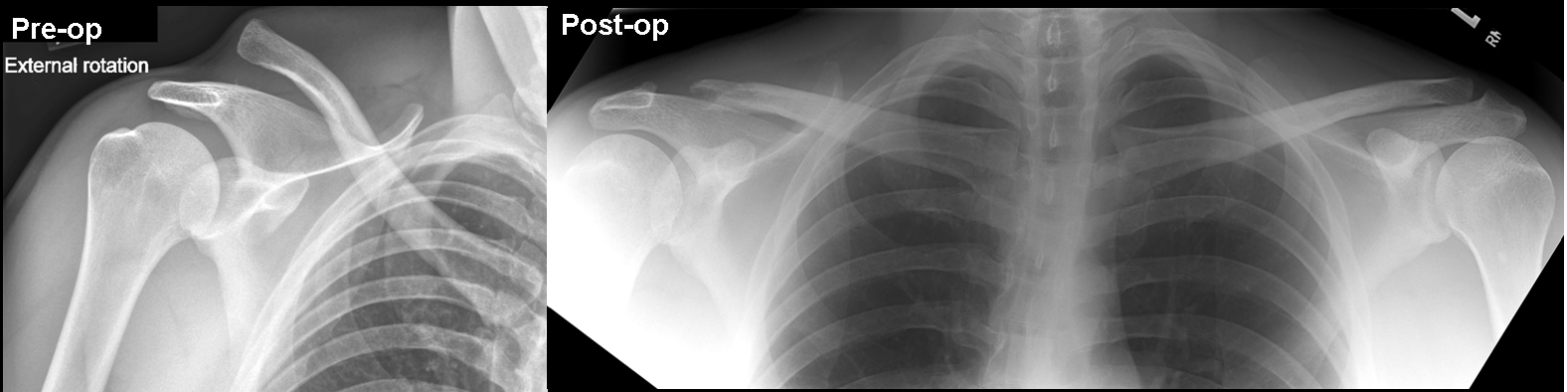
**Preoperative and postoperative X-rays**. Reprinted with permission: *OPERATIVE TECHNIQUES IN SPORTS MEDICINE, V12(1): 35–42, Millett PJ et al: "Acromioclavicular Joint Instability...". ^© ^2004 Elsevier Inc*

Radiographic evaluation included AP and axillary lateral views of the involved shoulder, both at initial and interval follow-up evaluations (Figure 2). Patients with evidence of glenohumeral arthritis, glenohumeral instability, impingement, or rotator cuff insufficiency, based on initial and follow-up history, physical and radiographic evaluation were excluded.

Patients' pain scores were recorded using a 10-point visual analog scale (VAS). Outcome rating scales included patient satisfaction with the procedure and the modified American Shoulder and Elbow Surgeons (ASES) shoulder score.[20] Shoulder strength testing was performed and graded on a scale of 0–5 using manual muscle testing, with 0 being no contraction of the muscle and 5 being normal.[20] A student's paired t-test was used to compare preoperative and postoperative outcome values. A p-value of 0.05 was used to define statistical significance.

### Docking Technique for AC Joint Reconstruction

**Figure 3:**
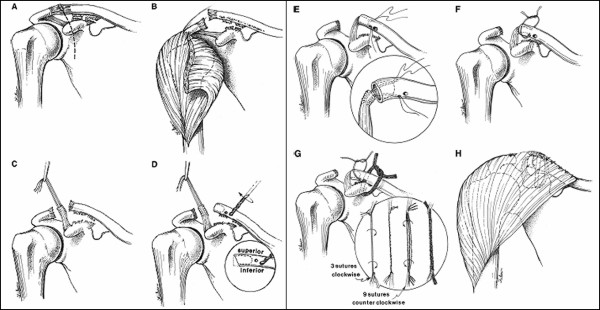
**Modified Weaver-Dunn technique for coracoclavicular ligament reconstruction**. (A) Initial Incision. (B) Deltoid exposure. (C) Coracoacromial (CA) ligament harvesting. (D) Preparation of clavicle. (E) CA ligament passage into clavicle. (F) Ligament transfer securing. (G) Absorbable suture that augments ligament transfer. (H) Completed repair.

**Figure 4:**
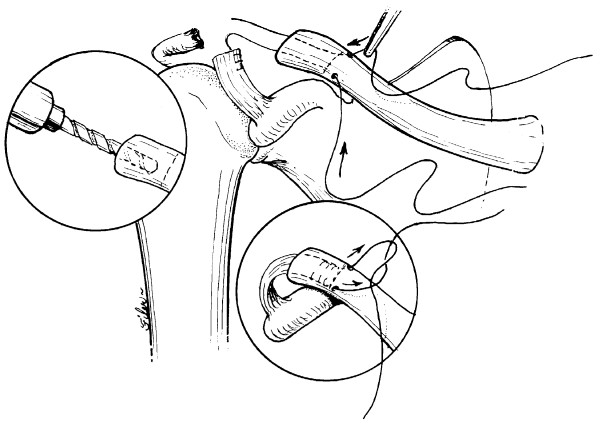
**AC reconstruction using the docking technique**. 4a & 4b reprinted with permission: *OPERATIVE TECHNIQUES IN SPORTS MEDICINE, V12(1): 35–42, Millett PJ et al: "Acromioclavicular Joint Instability...". ^© ^2004 Elsevier Inc*.

The procedure is performed under general anesthesia, supplemented by regional interscalene block for postoperative analgesia, with the patient in the beach-chair position. The incision is centered approximately 1.5 cm medial to the AC joint at the posterior aspect of the distal clavicle, and extends anterior-distally over the coracoid process (figure 3 and 4), which allows access to the CA ligament laterally and medially to the deltopectoral interval.[18]

The deltoid fascia has to be identified carefully. We prefer to take the deltoid down with a "hockey stick" incision through the deltopectoral interval medially and the deltotrapezial interval superiorly, in order to reflect the deltoid laterally. This facilitates visualization of the coracoid process. The deltoid is elevated with a full thickness flap over the mid-clavicle which extends laterally beyond the AC joint to the edge of the acromion. The deltoid can be reflected laterally saving the terminal branches of the axillary nerve.

Typically, the injury to the AC joint is readily apparent. The AC ligaments are split in a full thickness layer from medial to lateral with the deltotrapezial fascia and are preserved. Once the periosteal flap has been elevated from the clavicle with electrocautery and elevators, tagging sutures are placed at the medial apex which allows precise re-approximation of the deltopectoral interval during closure. Just medial to the tagging stitch, the incision is carried inferiorly through the deltopectoral interval to complete the "hockey stick" elevation of the deltoid visualizing the coracoid.

Once the deltoid has been lifted off the clavicle, the plane just superficial to the CA ligament should be identified and excess soft tissue should be carefully removed to define the borders of the CA ligament. By harvesting the reflected attachment of the CA ligament under the acromion with a scalpel, adequate length for the transferred CA ligament can be routinely obtained.

Transposing the CA ligament vertically in the shortest route superior to the clavicle determines the appropriate resection of the distal clavicle. At this location, typically 10–20 mm of the distal clavicle are resected. By harvesting the ligament in this method, and subsequently measuring for the resection, we have not had any instances of inadequate CA ligament length. We prefer to angle the clavicle osteotomy obliquely to leave a slightly more superior clavicle. This makes the ligament's turn into the medullary canal less abrupt.

The docking is performed by creating a 15 to 20 mm bone tunnel in the medullary canal of the distal clavicle. The deeper the medullary tunnel, the more surface area there is for ligament-to-bone healing. Drill holes for sutures are then created in the clavicle with a 2.0 mm drill bit exiting anteriorly and posteriorly approximately 15–20 mm medial to the end of the clavicle. A wide bone bridge prevents the sutures cutting through the bone.

The length of the CA ligament is now assessed. The clavicle should be manually reduced within 1 cm of the coracoid process. The CA ligament should be held to the end of the clavicle. The excess length of ligament should be equal to or just short of the length of the intramedullary tunnel. Ideally the CA ligament will reach the end of the medullary canal as it is reduced to within 5–10 mm of the coracoid process. This ensures optimal surface area for ligament to bone healing. If, however, the CA ligament is too long, it will bottom out in the tunnel and there will be inadequate tension to prevent recurrent deformity. If so, the ligament should be shortened appropriately. Once the measurements are accurate, the CA ligament is secured with 2 heavy, #2 nonabsorbable sutures (Fiberwire, Arthrex, Naples, FL), using Krakow type stitches. One limb of each suture from the CA ligament is then passed through each hole.

Prior to reducing the clavicle and tying the suture limbs over the superior cortex of the clavicle, the repair is augmented with PDS sutures around the coracoid. These act as internal splints until the transferred CA ligament heals securely. We have routinely used 9-strands of #1 PDS sutures wrapped into a cable.[18]

The PDS sutures are passed around the coracoid process, posterior to the pectoralis minor tendon insertion, and are then secured around the clavicle. This is accomplished with a curved wire suture passer (Linvatec, Largo, FL). Early in the series, 3 patients had additional augmentation with palmaris longus autografts. We now, however, routinely secure the repair with the absorbable 9 strand PDS cable alone.

Next, the clavicle is reduced and the CA ligament is "docked" into the medullary tunnel. The CA ligament is cycled to remove creep. Therefore the suture limbs are tensioned manually and the clavicle is carefully reduced. The suture limbs of the transferred CA ligament are then tied over the bony bridge superiorly on the clavicle. Since the displacing forces are significant and because some minor creep may occur, we recommend slight over-reduction of AC joint. It is important to make sure that the CA ligament is not of excessive length which would prevent adequate reduction of the distal clavicle. Once the transferred CA ligament is secured by tying the sutures over the bone bridge, the PDS sutures are tied.

The tensioning of the CA ligament is a critical step. The CA ligament should always be tensioned first using this technique. Appropriate reduction is achieved when the distal clavicle is within 5–10 mm of the coracoid process, depending on the patients' individual anatomy. If the PDS suture is tensioned before the CA ligament, the surgeon may falsely assume that the distal clavicle is adequately reduced and that the CA ligament has adequate tension. When the PDS sutures re-absorb over the ensuing weeks, the distal clavicle could displace as a result of the laxity in the transferred CA ligament. This technical error can be obviated using this 'docking' technique. The normal coracoclavicular distance varies from 11 to 13 mm.[21] We prefer to slightly over reduce the clavicle as a small amount of creep does occur, and over reduction insures that the clavicle will not displace superiorly. While it is conceivable that over reduction could cause impingement between the clavicle and coracoid, we have not experienced this in this series or in any of our patients with AC reconstructions.

The AC ligaments and trapezial fascia are then meticulously closed with heavy non-absorbable #2 suture. We bury the knots to prevent any irritation of the skin. Following wound closure, the patient is placed into a sling with a waist support (DonJoy, Vista, CA). The sling helps to elevate the proximal humerus and acromion, preventing additional stress on the reconstructed ligaments.

The presented technique varies from the originally described Weaver-Dunn technique by the way the CA ligament is transferred to the resected end of the clavicle in a bone-tunnel with the 'docking' technique and by using strong non-absorbable sutures (Fiberwire, Arthrex, Naples, FL). The original Weaver-Dunn technique used transosseous sutures. Additionally the reconstruction is secured by a PDS braid, that acts as a temporary internal splint.

### Postoperative Rehabilitation

The patients were immobilized for 4 weeks. Pendulums and passive motion started at 4 weeks time. Active and active-assisted motion commenced after the sixth week. Strengthening was typically delayed until 10–12 weeks after surgery. Sports were avoided for approximately 4 months.

## Results

The interval from injury to reconstruction averaged 11 months and ranged from 1 week to 6 years after the initial injury. Sixteen patients underwent a primary reconstruction and 2 patients underwent revision reconstruction after failed surgical treatments done elsewhere. Both the revision patients presented with symptomatic recurrence of the deformity and underwent a revision reconstruction of their AC instability. Both had intact CA ligaments, despite the operative notes stating that it had been harvested in the primary procedure. Early in this series, 3 of the reconstructions were augmented with a palmaris longus tendon autograft. With the numbers available, we could not demonstrate a difference between the group with and without autograft augmentation.

Preoperatively, all patients had aching shoulder pain, deformity and weakness that interfered with daily activities. Range of motion varied. Some of the patients in the chronic group had full active and passive motion but still complained of significant disability. Others in the acute injury group had loss of active range of motion. At the initial evaluation, active motion was limited in 9 patients, which contributed to the average elevation of 150° for the entire group (range, 90–180°). All patients had weakness in resisted forward elevation of the involved extremity when compared to the asymptomatic contralateral side.

Pre- and postoperative active range of motion was compared. Eight patients had a full range of motion before and after surgery (47%), nine had limited active motion before the surgery. This group with limited preoperative motion had an average forward elevation of 135° and an external rotation with the arm at maximal abduction averaging 61°. Four of these 9 patients obtained full range of motion after the surgery. The remaining 5 patients gained significant range of motion after the surgery, averaging 155° of forward elevation and 70° of external rotation with the arm maximally abducted. Thus, range of motion improved significantly (p = <0.0001) in all patients with preoperative limited range of motion.

Likewise, there was a consistent and significant postoperative strength gain among all patients, clinically assessed with a mean grade of 4/5 on preoperative evaluations. Normal strength grade of 5/5 was achieved in 16 of 17 patients (94%) at final follow-up evaluation.

All patients reported significant improvement of their subjective pain scores from a preoperative median of 7/10 to a median of 0/10 at final follow-up. Likewise, all patient's ASES score improved postoperative from a mean baseline of 46 to 86 (p < 0.02), including the 1 patient with recurrence of deformity.

More than 50% superior displacement of the distal clavicle defined recurrence of deformity. Follow-up radiographs revealed maintenance of reduction in 16 of the 17 patients (94%), with only 1 of 17 (6%) sustaining a recurrence of the deformity (100% displacement). This patient was seen 8 weeks post-op without any deformity. He subsequently returned to karate, against medical advice, and at 3 months postoperatively presented with a recurrence of the deformity. He was followed clinically and did well by other objective outcomes measures.

There was no significant difference in outcome when comparing those treated acutely (less than 4 weeks from the time of the injury: n = 8, mean = 2 weeks, range 3 days to 4 weeks) versus those treated for a chronic injury (n = 9, mean = 16 months, range 2–60 months).

There were no infections, or other types of short or long-term complications in the series. No patients had atrophy of the deltoid and there were no deltoid detachments. All patients were able to return to their pre-surgical occupation following surgery. Of the 3 patients who suffered AC injuries at work and had associated workers' compensation claims, all were able to return to work at the same level as before the injury. Of the 8 patients who competed in recreational sports prior to their injury, all were able to return to sport at the same level. There were no college or professional athletes in this series.

## Discussion

Since Cooper's first description of surgical fixation of an AC dislocation in 1861, [22] many techniques, often in combination, have been described to address AC separations. The Weaver-Dunn procedure remains one of the main surgical techniques performed world wide – combining a lateral clavicle resection as described by Morestin, [9] Mumford, [10] and others, [23-28] with a transfer of the CA ligament similar to what Cadenat [29] described in 1917.

This study was performed to evaluate the preliminary results of a modified technique to treat complete AC joint separations. When compared to the literature, our series fares better in terms of maintenance of reduction and functional gains. [15,23-28,30,31] Objectively, our technique identifies 2 key points that are different from the traditional Weaver-Dunn modifications, that may explain the better results. The classic Weaver-Dunn reconstruction, as described in 1972 and whose results have been proven by many over the years, [32-36] provides the basis for our technique with 2 modifications: 1) a CA ligament 'docking' technique using strong non-absorbable sutures. 2) Placement of a PDS augmentation suture braid, as originally described by Noble, [19] but tightened only after the CA transfer has been docked and tied into place. This augmentation is in accordance with the growing consensus that has formed towards protecting the construct while the CA ligament is healing. [3,15,37-40]

The term "docking technique" was coined in 2000 [16,17] to describe a reconstruction of the elbow MCL, using a similar procedure for excellent ligament tensioning and tendon-to-bone integration. The authors believe this is critical to achieving a good outcome.

In this series, the clavicle was successfully maintained in a reduced position in 16 of 17 patients, while there were also statistically significant and clinically relevant improvements in shoulder range of motion, strength and pain. Our radiographic results are comparable to the best outcomes published in the English literature for AC reconstruction with a CA ligament transfer. Guy [40] obtained maintenance of reduction in 22 of 23 (96%) patients at final follow up with a modification of the Weaver-Dunn technique augmented with a CC screw. Therefore an additional outpatient procedure for removal was required. Although our follow-up is shorter, the only loss of reduction was seen at 12 weeks in a patient that returned to contact sports against medical advice. The only failure of reduction in Guy's study was seen right after hardware removal. This supports our impression, that the ligament transfer is healed after approximately 12 weeks. In contrast, Weaver and Dunn's originally reported 20% recurrence rate. This rate varies in the literature between 15–25% depending on the series consulted. [15,23-28,30,31] For the patients in our series, all were able to return to recreational sport and to work. Early in the series we did use palmaris longus autografts in 3 patients but do not use this routinely in our current treatment algorithm. In collision athletes or revision settings with possible poor quality of the CA ligament, we do consider the use of autograft or allograft supplementation.

While a limitation of the study is the small size of the series and the lack of long-term follow-up, the study still provides useful information about the technique's outcomes. The Weaver Dunn technique is widely recognized as a standard treatment but many key steps are not discussed in detail in most publications.

The docking technique is an excellent way to achieve appropriate tension on the transferred CA ligament (reducing the clavicle within 5 to 10 mm of the coracoid) with maximized potential for solid ligament-to-bone integration. The technique's limitations are minimized compared to the original Weaver-Dunn procedure. A major concern is to neutralize the high forces postoperatively that may put the transferred ligament in danger. The in-growth of the transferred CA ligament in the bone tunnel is protected temporarily by the strong PDS cable. We believe that the results of this study, with its optimized technique, will serve as a useful reference as other more anatomic or less-invasive techniques are developed.

In many instances, patients with AC separations are treated surgically if they fail initial conservative treatment or if they involve severe dislocations such as Rockwood types IV, V, or VI. For clarification, we consider an AC dislocation to be a type V, if there is superior displacement of the distal clavicle greater than 100% that cannot be passively reduced by a manually applied upward force on the proximal humerus and acromion. We believe that soft tissue blocks the reduction and portends a worse outcome with non-operative treatment. The rationale behind a successful surgical approach is to restore stability to the distal clavicle effectively resuming its role in suspending the scapula, aiding to support the upper extremity weight. This study hints at the value of AC joint restoration in both early surgical treatment of acute severe injuries (5 patients intervened less than 2 weeks from injury) as well as chronic dislocations that represented failures of conservative (10 patients) and surgical treatment (2 patients), although the study was not appropriately powered to show a difference in outcomes for chronic versus acute reconstruction The results of this study suggest that previous success rates can be matched and even improved using the docking technique modifications to reconstruct the AC joint.

## Competing interests

Financial competing interests

Peter J. Millett discloses a financial relationship with Arthrex but it is unrelated to this manuscript. All authors declare that they have no competing interests.

## Authors' contributions

All authors made substantive intellectual, conception and design contributions to this study. PJM was the primary investigator. He initiated and conceptually designed the study; he performed all surgeries and took part in the data collection. He drafted the manuscript. SB made substantial contribution to data analysis and interpretation, revised the manuscript critically and gave approval of the final version. RB was taking part in study design, data acquisition, analysis, interpretation, drafting and gave final approval of the manuscript. IHP was taking part in study design, data acquisition, analysis, interpretation, drafting and gave final approval of the manuscript.

## Pre-publication history

The pre-publication history for this paper can be accessed here:


